# Medical and sociodemographic factors predict persistent smoking after coronary events

**DOI:** 10.1186/s12872-017-0676-1

**Published:** 2017-09-06

**Authors:** Elise Sverre, Jan Erik Otterstad, Erik Gjertsen, Lars Gullestad, Einar Husebye, Toril Dammen, Torbjørn Moum, John Munkhaugen

**Affiliations:** 1 Department of Medicine, Drammen Hospital, PB 800, 3004 Drammen, Norway; 2 Department of Behavioural Sciences in Medicine, University of Oslo, Oslo, Norway; 3 Department of Medicine, Vestfold Hospital, Tønsberg, Norway; 4 Department of Cardiology, Oslo University Hospital Rikshospitalet, The Faculty of Medicine, University of Oslo, Oslo, Norway

**Keywords:** Smoking, Smoking cessation, Coronary heart disease (CHD), Secondary prevention, Sociodemographic factors, Medical risk factors, Psychosocial risk factors

## Abstract

**Background:**

Understanding the determinants of persistent smoking after a coronary event constitutes the basis of modelling interventions of smoking cessation in secondary prevention programs. We aim to identify the potentially modifiable medical, sociodemographic and psychosocial factors, comprising the study factors, associated with unfavourable risk factor control after CHD events.

**Methods:**

A cross-sectional explorative study used logistic regression analysis to investigate the association between study factors and smoking status in 1083 patients hospitalized with myocardial infarction and/or coronary revascularization. Hospital record data, a self-report questionnaire, clinical examination and blood samples were applied.

**Results:**

At the index hospitalization, 390 patients were smoking and at follow-up after 2–36 months 167 (43%) of these had quit, while 230 reported persistent smoking. In adjusted analyses, unemployed or disability benefits (Odds ratio (OR) 4.1), low education (OR 3.5), longer smoking duration (OR 2.3) and not having ST-elevation myocardial infarction (STEMI) as index event (OR 2.3) were significantly associated with persistent smoking. Psychosocial factors at follow-up were not associated with persistent smoking. Smokers reported high motivation for cessation, with 68% wanting help to quit. Only 42% had been offered nicotine replacement therapy or other cessation aids. Smokers rated use of tobacco as the most important cause of their coronary disease (6.8 on a 1–10 Likert scale).

**Conclusions:**

Low socioeconomic status, prior duration of smoking, and not having STEMI as index event were associated with persisting smoking. Persistent smokers in this study seem to have an acceptable risk perception and were motivated to cease smoking, but needed assistance through cessation programs including prescription of pharmacological aids.

**Trial registration:**

Registered at ClinicalTrials.gov: NCT02309255, registered retrospectively.

## Background

The causal role of cigarette smoking in the development and progression of coronary heart disease (CHD) is overwhelmingly documented [1, 2]. Smoking is the leading avoidable cause of death in the developed world [3] and increases the risk of coronary events [4] and total mortality by up to 50% [1, 4]. Smoking thus remains the single most important cardiovascular risk factor to modify in order to improve prognosis in CHD patients [2–4].

Different smoking cessation programs and pharmacological treatment [5–7] increase the likelihood of cessation in clinical studies. Still, clinical practice across Europe suggests that about half of the daily smokers surviving a coronary event continue smoking [8]. While the prevalence of daily smoking in the general European population decreased substantially over the past decade [9], only a modest reduction (20% to 16%) was seen in CHD patients over the past 20 years in the EuroAspire studies [8, 10]. In the US, the reduction in the number of daily smokers from 1980 to 2000 was 12% in the general population compared to 5% in CHD patients [11]. It is concerning that the prevalence of daily smoking actually has increased among the youngest CHD patients [10].

A complex array of patient and healthcare factors influence smoking behaviour in coronary patients [2, 3, 12–17]. Identification of potentially modifiable medical and psychosocial factors associated with persistent smoking after coronary events could be important for the development of individually tailored interventions of smoking cessation with sustained effect [2, 5, 7, 18].

The NORwegian-CORonary (NOR-COR) Prevention Study identifies sociodemographic, medical, and psychosocial factors, comprising the study factors, associated with unfavourable risk factor control after CHD events (phase I). Moreover, the project aims to target the study factors of importance for risk factor control in tailored interventions (phase II) [18]. The present exploratory analysis aims to identify the study factors associated with persistent smoking in a cross-sectional survey.

## Methods

### Design and population

The design, methods, and baseline characteristics of the NOR-COR Study have been described elsewhere [18]. A cross-sectional study was conducted at two general Norwegian hospitals (Drammen and Vestfold) with a total catchment area of 380,000 inhabitants, corresponding to 7.4% of the Norwegian population. In total, 1789 consecutive patients aged 18–80 years with a first or recurrent coronary event/treatment (i.e. acute myocardial infarction, coronary artery by-pass graft operation, and/or percutaneous coronary intervention) were identified from hospital discharge lists over the three years (2011–14) prior to study inclusion. The index event was defined as the last coronary event prior to inclusion. Of the identified patients, 423 were excluded due to cognitive impairment (*n* = 28), psychosis (*n* = 18), drug abuse (*n* = 10), short life expectancy (*n* = 136), death (*n* = 160), not being able to understand Norwegian language (*n* = 44), and other (*n* = 26). Of the remaining 1366 eligible patients, 1127 (83%) consented to participate in attending a clinical visit and completing a comprehensive questionnaire [18] at 2–36 months follow-up after the index event. Smoking status at follow-up was missing in 44 patients, leaving 1083 in the final analyses. As smoking status at the index event was missing in seven patients who reported daily smoking at follow-up, there is thus a minor discrepancy between the proportion of smokers reported at index event and at follow-up.

All participants gave informed consent before study participation. The NOR-COR study was approved by the Regional Committee of Ethics (REK Sør-Øst) 12. February, 2014 (2013/1885).

### Outcome assessment

The primary outcome variable was smoking status at follow-up compared to smoking status at the index event, categorized as persistent smoker vs. quitter. Smoking status at index event was recovered from hospital medical records. Patients who smoked at the time of the index event were categorized as current smokers. Smoking at follow-up was recorded from the self-report questionnaire. All patients who reported daily smoking [cigarettes (*n* = 225), pipes (*n* = 0) or cigars (*n* = 5)] were categorized as persistent smokers.

### Covariates (study factors)

#### Study data registered at the time of the index event

Demographic variables, risk factors, somatic comorbidity summarized according to the Charlson comorbidity index [19] and information about the index coronary event and treatment were registered from hospital medical records [18].

#### Study factors registered at follow-up 2–36 months after the index event

Sociodemographic factors included marital status, education, and employment status. Medical factors included coronary risk factors and cardiovascular medication. Psychosocial factors included anxiety and depression (Hospitality Anxiety and Depression Scale) [20], Type D personality [21], worry (Penn State Worry Questionnaire) [22], insomnia (Bergen Insomnia Scale) [23], illness perception (Brief illness perception questionnaire) [24] and perceived risk [25]. Moreover, information about smoking behaviour, motivation and treatment needs were obtained by the self-report questionnaire [18]. Participation in cardiac rehabilitation programs was based on hospital medical records, including lists from the cardiac rehabilitation departments.

### Statistics

Using SPSS version 21, data were analysed in a cross-sectional design with the study factors as the main exposure variables and smoking status (persistent smokers vs. quitters) at follow-up as the main outcome variable. The distribution of study factors according to smoking status (never smoker, former smoker, current smoker) at the index event and at follow-up (persistent smokers, quitters) were reported as frequencies and percentages. A forward, stepwise binary logistic regression analysis was used to calculate crude and multi-adjusted odds ratio (OR) and 95% confidence intervals (CI) for study outcomes with interaction terms between independent variables as indicated. The level of significance was set to *p* < 0.05. Covariates with *p*-values between 0.05 and 0.1 in crude analyses were also selected as candidates for the multivariate analyses since more traditional levels (i.e. 0.05) can fail to identify variables that might turn out to be significant when actually included in the adjusted models.

## Results

Characteristics of the study population at the time of the index event are presented in Table 1. Mean age was 62 (SD 10) years and 21% were women. A total of 390 (36%) patients were smoking at the time of the index event. Compared to former and never smokers, the group of current smokers consisted of more females than males, and were characterized as a group by their younger age, lower education levels, more often having ST-elevation myocardial infarction (STEMI) as index event as well as having fewer previous coronary events.

**Table 1:** Patient characteristics according to smoking status at the index coronary event

Study factors	Never smoker (*n* = 250)	Former smoker (*n* = 436)	Current smoker (*n* = 390)
Sociodemographic factors			
Age at index event, mean (SD)	63.3 (10.3)	63.2 (8.7)	58.5 (9.5)
Number of months since the index event, mean (SD)	16.5 (10.4)	16.5 (10.2)	18.2 (10.9)
Female sex, n (%)	51 (20.4)	79 (18.1)	97 (24.9)
Ethnic minority background, n (%)	12 (4.8)	8 (1.8)	11 (2.8)
Low education, n (%)	156 (62.4)	291 (66.7)	296 (75.9)
Medical factors			
*Coronary index event and treatment:*			
ST-elevation infarction, n (%)	54 (21.6)	102 (23.4)	167 (42.8)
Non-ST-elevation infarction, n (%)	137 (54.8)	218 (50.0)	182 (46.7)
Stable or unstable angina, n (%)	59 (23.6)	116 (26.6)	44 (11.3)
More than 1 coronary event, n (%)	71 (28.4)	151 (34.6)	97 (24.9)
*Comorbidity:*			
Charlson co-morbidity sum score, mean (SD)	4.0 (1.3)	4.3 (1.5)	3.9 (1.4)
Heart failure, n (%)	21 (8.4)	65 (14.9)	54 (13.8)
Stroke or transient ischemic attack, n (%)	14 (5.6)	39 (8.9)	24 (6.2)
Peripheral artery disease, n (%)	12 (4.8)	39 (8.9)	42 (10.8)
Chronic obstructive pulmonary disease, n (%)	2 (0.8)	45 (10.3)	47 (12.1)
Hypertension, n (%)	129 (51.6)	231 (53.0)	249 (63.8)
Diabetes, n (%)	35 (14.0)	92 (21.1)	49 (12.6)
Treatment at hospital discharge			
Aspirin, n (%)	247 (98.8)	425 (97.5)	387 (99.2)
Additional antiplatelet therapy, n (%)	212 (84.8)	373 (85.6)	360 (92.3)
Statins, n (%)	239 (95.6)	414 (95.0)	379 (97.2)
ACEI^a^ or ARBs^b^, n (%)	141 (56.4)	249 (57.1)	207 (53.1)
Beta-blockers, n (%)	212 (84.8)	373 (85.6)	329 (84.4)

^a^ACEI, angiotensin converting enzyme inhibitor

^b^ARB angiotensin receptor blocker

Sociodemographic, medical, and psychosocial factors in persistent smokers and quitters at follow-up 2–36 (mean 16) months after the index event are shown in Table 2. At follow-up, 167 (43%) of the registered smokers at the index event had quit smoking, while 230 patients reported persistent smoking. Almost all persistent smokers had been smoking for 20 years or more, and 60% had been smoking for more than 40 years. Seventy-three percent of the persistent smokers reported having reduced their cigarette use since the index event. In bivariate analyses, low education, living alone, unemployed or disability benefit, longer smoking duration, more than one coronary event prior to the index event, non-participation in cardiac rehabilitation, not having STEMI as index event, low physical activity, and no prescription of statins were significantly more prevalent in persistent smokers than in quitters. No significant differences in psychosocial factors were found between persistent smokers and quitters. These findings were consistent in sub-group analyses by age and gender.

**Table 2:** Sociodemographic, medical, and psychosocial factors in smokers and quitters at follow-up^a^ after the coronary event

Study factors	Quitted after the index event (*n* = 167)	Persistent smokers (*n* = 230)	*p-*value
Sociodemographic factors			
Age at index event, mean (SD)	57.7 (9.4)	59.3 (9.3)	*p* = 0.05
Number of months since the index event, mean (SD)	16.8 (10.9)	18.9 (10.8)	*p* = 0.05
Female sex, n (%)	38 (22.8)	56 (24.3)	*p* = 0.71
Ethnic minority background, n (%)	4 (2.4)	10 (4.3)	*p* = 0.30
Living alone, n (%)	28 (16.8)	57 (24.8)	*p <* 0.05
Low education, n (%)	116 (69.5)	189 (82.2)	*p* < 0.001
Unemployed or on disability benefits, n (%)	33 (19.8)	85 (37.0)	*p* < 0.001
Medical factors			
Duration of smoking, years, n (%)			
< 20	11 (6.6)	8 (3.5)	*p* < 0.001
20–39	99 (59.3)	64 (27.8)	
> 40	53 (31.7)	137 (59.6)	
ST-elevation infarction/non ST-elevation infarction and angina, n (%)	78 (46.7)/ 89 (53.3)	84 (36.5)/ 146 (63.5)	*p* < 0.05
More than 1 coronary event, n (%)	30 (18.0)	77 (33.5)	*p* < 0.001
Participation in cardiac rehabilitation, n (%)	95 (56.9)	103 (44.8)	*p* < 0.05
Charlson co-morbidity sum score, mean (SD)	3.8 (1.3)	4.0 (1.4)	*p* = 0.14
Use of antiplatelets at follow-up, n (%)	164 (98.2)	221 (96.1)	*p* = 0.22
Use of statins at follow-up, n (%)	160 (95.8)	207 (90.0)	*p <* 0.05
Low physical activity^b^, n (%)	93 (55.7)	186 (80.9)	*p* < 0.001
Body Mass Index >30 kg/m^2^, n (%)	61 (36.5)	58 (25.2)	*p* < 0.05
Low density lipoprotein cholesterol >1.8 mmol/l, n (%)	88 (52.7)	124 (53.9)	*p* = 0.66
Blood pressure > 140/90 (140/80 diabetes) mmHg, n (%)	60 (35.9)	74 (32.2)	*p* = 0.74
Diabetes, n (%)	16 (9.6)	36 (15.7)	*p* = 0.08
Psychosocial factors			
Hospital Anxiety and Depression Score - depression ≥11, n (%)	12 (7.2)	13 (5.7)	*p* = 0.61
Hospital Anxiety and Depression Score - anxiety ≥11, n (%)	22 (13.2)	27 (11.7)	*p* = 0.81
Type D personality disorder, n (%)	41 (24.6)	56 (24.3)	*p* = 0.89
Worry score (PSWQ^c^), mean (SD)	40.1 (13.9)	40.3 (13.9)	*p* = 0.87
Insomnia^d^, n (%)	80 (47.9)	114 (49.6)	*p* = 0.65

^a^2–36 months after the index coronary event

^b^Physical activity less than 30 min of moderate activity 2–3 times weekly

^c^Worry was assessed by the Penn State Worry Questionnaire (PSWQ), a 16 item measure of pathological worry

^d^Measured by Bergen insomnia Scale

The persistent smokers reported a high motivation (average 7.8 on a 1–10 Likert scale) for smoking cessation and only 14% reported low (≤3) motivation. Sixty eight percent wanted help to quit smoking. However, only 42% reported having been offered nicotine replacement therapy or any other form of cessation aid. In total, 35% of the persistent smokers and 27% of the quitters (*p* = 0.14) reported having no current follow-up for their CHD in primary or specialist healthcare. Smoking was rated as the most important cause of CHD by both persistent smokers and quitters, but quitters rated the importance of smoking as risk factor higher (7.5 vs. 6.8 on a 1–10 Likert scale, *p* < 0.05). Persistent smokers felt they had a higher likelihood of having a new heart attack within the next 12 months than quitters (3.3 vs. 2.5 on a 1–10 Likert scale, *p* < 0.01). They also felt they could do less to help reduce that risk (6.4 vs. 7.0 on a 1–10 Likert scale, p < 0.05). Compared to quitters, persistent smokers thought they would have to restrict their daily activities more in the long-term (3.0 vs. 4.2 on a 1–10 Likert scale, *p* < 0.001). Persistent smokers scored significantly lower on treatment control compared to quitters (7.8 vs. 6.6 on a 1–10 Likert scale, *p* < 0.001), while no other differences in illness perception were found. A considerable subgroup of both persistent smokers (21%) and quitters (13%) perceived “no need to change lifestyle”.

The odds ratios for persistent smoking compared to quitting smoking after the index event by study factors are shown in Table 3. In crude analyses, unemployment or disability benefits were the strongest predictors of persistent smoking, followed by longer duration of smoking, low education, >1 coronary event, living alone, no participation in cardiac rehabilitation and not having STEMI as index event. In multi-adjusted analyses the study factors significantly associated with persistent smoking were: unemployment or disability benefits, low education, longer duration of smoking, and not having STEMI as index event.

**Table 3:** Odds ratios for persistent smoking after the coronary index event calculated with logistic regression analysis

Study factors	Model 1 (OR, 95% CI)	*p-*value	Model 2 (OR, 95% CI)	*p*-value
	Crude^a^		Multi-adjusted^b^	
Sociodemographic factors				
Mean age at index event (OR per year)	1.02 (1.00–1.04)	*p* = 0.05	0.97 (0.90–1.03)	*p* = 0.27
Time since the index event (OR per year)	1.02 (1.00–1.04)	*p* = 0.05	1.01 (0.98–1.05)	*p =* 0.41
Female gender	1.09 (0.68–1.75)	*p* = 0.71	2.17 (0.85–5.52)	*p =* 0.10
Living alone	1.69 (1.01–2.82)	*p* < 0.05	1.23 (0.48–3.11)	*p =* 0.67
Low education	2.20 (1.36–3.57)	*p* = 0.001	3.35 (1.43–7.81)	*p <* 0.01
Unemployed or on disability benefits	3.01 (1.81–5.02)	*p <* 0.001	4.12 (1.80–9.41)	*p =* 0.001
Medical factors				
Not having ST-elevation infarction as index event	1.53 (1.02–2.29)	*p* < 0.05	2.30 (1.08–4.40)	*p <* 0.05
More than 1 coronary event	2.30 (1.42–3.72)	*p* = 0.001	1.53 (0.63–3.72)	*p =* 0.35
Participation in cardiac rehabilitation	0.62 (0.41–0.92)	*p* < 0.05	0.78 (0.38–1.60)	*p =* 0.50
Charlson co-morbidity sum score	1.12 (0.96–1.32	*p* = 0.14		
Duration of smoking (years)	2.93 (1.62–2.71)	*p* < 0.001	2.34 (1.41–3.88)	*p =* 0.001
Psychosocial factors				
Hospital Anxiety and Depression Score - total > 11	1.06 (0.70–1.62)	*p* = 0.78		
Type D personality	1.03 (0.65–1.65)	*p* = 0.89		
Worry score (PSWQ^c^)	1.00 (0.99–1.01)	*p* = 0.87		
Insomnia^d^	1.10 (0.73–1.65)	*p* = 0.65		
*Perceived risk (1–10 Likert scale)*				
What do you feel is the likelihood of having a new heart attack over the next 12 months?	1.15 (1.05–1.25)	*p* < 0.01	1.01 (0.86–1.18)	*p =* 0.93
How much do you feel you can help reduce your risk of having another heart attack?	0.91 (0.84–0.99)	*p* < 0.05	0.88 (0.76–1.02)	*p =* 0.09
How much do you think you will have to restrict your activities in the long-term du to your heart condition?	1.17 (1.08–1.27)	*p* < 0.001	1.00 (0.87–1.17)	*p =* 0.90
*Brief Illness Perception Questionnaire (1–10 Likert scale)*				
How much does your illness affect your life? (consequences)	0.99 (0.92–1.06)	*p =* 0.76		
How long do you think your illness will continue? (timeline)	0.98 (0.91–1.04)	*p =* 0.45		
How much control do you feel you have over your illness? (personal control)	0.95 (0.88–1.02)	*p =* 0.16		
How much do you think your treatment can help you? (treatment control)	0.80 (0.72–0.88)	*p* < 0.001	0.88 (0.75–1.02)	*p =* 0.09
How much do you experience symptoms from your illness? (identity)	0.83 (0.92–1.07)	*p =* 0.83		
How concerned are you about your illness? (concern)	0.98 (0.93–1.06)	*p =* 0.74		
How well do you feel you understand your illness? (understanding)	0.97 (0.89–1.05)	*p =* 0.41		
How much does your illness affect you emotionally? (emotional response)	0.96 (0.90–1.02)	*p =* 0.19		

Quitted smoking after the index event is the reference category

^a^Model 1, crude analyses

^b^Model 2, multi-adjusted with including all variables with *p* < 0.1 in crude analysis (adjusted for all variables included in the model)

^c^Worry was assessed by the Penn State Worry Questionnaire (PSWQ), a 16 item measure of pathological worry

^d^Measured by Bergen insomnia Scale

## Discussion

Almost 60% of patients in routine clinical practice persisted smoking 2–36 months after a coronary event. In particular, low socioeconomic status, longer duration of smoking and not having STEMI as index event were associated with persistent smoking in multi-adjusted analyses, while psychosocial factors and participation in cardiac rehabilitation were not. Almost all persistent smokers had been smoking for a long time, with 60% having been smokers for more than 40 years. Most persistent smokers seemed to be aware of the risk associated with smoking and reported a high motivation for cessation. More than 70% of persistent smokers had reduced the number of cigarettes smoked after the index event. However, only 42% reported having been offered any smoking cessation aids, and 35% had no current follow-up in primary or specialist healthcare for their CHD.

Low education, unemployment and the claiming of disability benefit s were the factors most strongly associated with persistent smoking, concurring to the well-established inverse association between smoking and socioeconomic status [2, 3, 26]. Even though socioeconomic position is not easily modified, our findings suggest that these patients should be systematically identified during hospitalisation for coronary events and offered smoking cessation programs.

In the present study, STEMI as the coronary index event was significantly associated with quitting smoking. A qualitative study finding that the urgency of managing STEMI bolsters the impression of suffering a life-threatening event could be relevant in this context since such a perception may increase the patient’s motivation and willingness to change lifestyle [27]. In contrast, the initial uncertainty of the diagnosis in non ST-elevation myocardial infarction (NSTEMI) and unstable angina pectoris may lead to reduced appreciation of the seriousness of the condition [27]. The majority of persistent smokers in the present study, however, appear to be aware of the risk associated with smoking, with smoking rated as the most important cause of CHD.

Most persistent smokers reported a high motivation for smoking cessation and 73% reported having reduced their smoking since the coronary event, while almost 70% reported that they wanted immediate assistance with cessation. Even though we have not measured the different stages of change [28], this suggests that these patients have passed the pre-contemplation stage (i.e. no intention to quit). By comparison, 50% of CHD patients smoking at follow-up had passed the pre-contemplation stage in the EuroAspire III study. As recommended in clinical guidelines [2], motivation and readiness for smoking cessation should be assessed in all patients admitted to hospital for coronary events. A long smoking history, the reported reduction in consumption of cigarettes and the high level of motivation for cessation without success suggest substantial nicotine dependency. Nicotine dependency is a well-known barrier to smoking cessation [12, 14], and the longer the duration of smoking, the less likely is a change in smoking behaviour [28]. It is of concern, therefore, that only 42% of the persistent smokers in the present study had been offered cessation aid, which is in accordance with other studies of CHD patients [29, 30]. Smoking receives less attention from cardiologists than other risk factors [12], possibly because physicians believe themselves to possess limited intervention skills in behavioural counselling [16]. It may also reflect a reluctance among health professionals to believe that their patients have the ability to quit smoking. This may partly explain why smoking cessation therapy is not offered as indicated [16].

Surprisingly, none of the measured psychosocial factors (anxiety, depression, worry, insomnia, type D personality) differed between quitters and persistent smokers on average 1.7 years after the index coronary event. Our findings are contrary to studies reporting that individuals with depression are more likely to smoke and less likely to quit smoking successfully [31, 32]. Other studies have found no association between persistent smoking and either depression or anxiety [33]. The frequency of smoking in patients with type D personality is higher than in patients without type D in some studies [34], while others have found no differences [35]. The level of psychosocial distress decreases with increasing time since the coronary event [36] and the timing of assessment of psychosocial factors may explain the conflicting association between smoking and psychosocial factors observed in previous studies. Variation in measurement methods used for evaluation of psychological symptoms [32, 37] is another possible explanation. Psychosocial factors were not measured at the index event and we therefore do not know if these factors predict persistent smoking at follow up.

In accordance with two randomized clinical trials [38, 39], we found no effect of participation in cardiac rehabilitation on smoking cessation in adjusted analyses. The most recent Cochrane review on cardiac rehabilitation programs [40] did not address the effect of cardiac rehabilitation on smoking cessation, while the 2004 review [41] found a small positive effect of cardiac rehabilitation on cessation. Better smoking cessation interventions are urgently needed in clinically implemented cardiac rehabilitation programs [2].

A Cochrane review of tobacco treatment trials found that intensive counselling, initiated during hospitalization, significantly increased quit rates at 12 months follow-up [7]. Adding nicotine replacement therapy further increased quitting compared with counselling alone [7, 17]. The small reduction in daily smoking in CHD patients across Europe over the past few decades [8, 10], however, substantiates the need of novel and better strategies to ensure the implementation of evidenced based cessation programs in clinical practice. Despite a long and heavy smoking history, most patients in this cohort seem to be aware of the risk associated with smoking and were motivated to quit smoking. Hospitalization for a coronary event provides an important opportunity for quitting smoking and the chance of successful smoking cessation has recently been shown to be higher with immediate [42] and abrupt [43] quitting. Effective, proactive counselling tailored to the readiness to quit smoking should therefore be the standard of care for managing all smokers during hospitalization for the index coronary event [17]. Furthermore, systematic referral to outpatient smoking cessation programs adapted to each patient’s profile and needs [18] and routinely prescribing pharmacological aids may further facilitate cessation. High risk sub-groups with low socioeconomic status, a long history of smoking and those having less dramatic coronary events such as NSTEMI or angina, are at increased risk of persistent smoking and should receive particular attention and be considered for extended follow-up.

### Study limitations and strengths

Smoking and other important study factors were measured by self-reporting, and are thus prone to measurement errors and recall bias. Additional confounders should be considered. Data for the date of smoking cessation, the use of cessation aids among quitters and specific questionnaires addressing nicotine dependence were not available. We do not have enough power to perform multi-adjusted analyses in sub-groups of age and gender, but no important differences in the major study factors were found in unadjusted sub-group analyses. High participation rate (83%), the routine clinical setting and few missing data are important strengths of the study. A reproducibility study of the NOR-COR questionnaire demonstrated highly acceptable test-retest values for all key items and instruments [44].

## Conclusion

Low socioeconomic status, prior duration of smoking, and not having STEMI as index event were associated with persistent smoking in coronary patients, while psychosocial factors and participation in cardiac rehabilitation were not. A majority of persistent smokers appeared to be aware of the risk associated with smoking and were motivated for smoking cessation. Given the well-documented benefit of smoking cessation, there is considerable potential for better interventions to facilitate cessation in CHD patients, including systematic referral to cessation programs including prescription of pharmacological aids.

## Abbreviations

ACEI: Angiotensin converting enzyme inhibitor
ARB: Angiotensin receptor blocker
CHD: Coronary heart disease
CI: Confidence interval
NSTEMI: Non-ST elevation infarction
OR: Odds ratio
PSWQ: Penn State Worry Questionnaire
SD: Standard deviation
STEMI: ST-elevation myocardial infarction
